# Growth Patterns of Children of Same Geographic Background Reared in Different Environments

**DOI:** 10.4274/jcrpe.1612

**Published:** 2014-12-05

**Authors:** Sevil Arı Yuca, Yaşar Cesur, Selim Kurtoğlu, Mustafa Mümtaz Mazıcıoğlu, Emine Ayça Cimbek

**Affiliations:** 1 Selçuk University Faculty of Medicine, Department of Pediatric Endocrinology, Konya, Turkey; 2 Bezmialem Foundation University Faculty of Medicine, Department of Pediatric Endocrinology, İstanbul, Turkey; 3 Erciyes University Faculty of Medicine, Department of Pediatric Endocrinology, Kayseri, Turkey; 4 Erciyes University Faculty of Medicine, Department of Family Medicine, Kayseri, Turkey

**Keywords:** growth, children, weight, height, percentile

## Abstract

**Objective:** Growth charts are essential tools used to assess children’s health status. The aim of the present study was to determine the effect of environmental factors on the growth of children of a common geographic background. We constructed growth charts for children living in the East of Turkey and compared them with those for Turkish children living in other regions or countries.

**Methods:** Growth data were obtained from 6 917 school children living in Eastern Turkey. The median values on smoothed percentile curves for the study subjects were compared with those for Turkish children living in the West of Turkey, in Western Europe and in Germany.

**Results:** Children living in Turkey were lighter than their European peers at early ages. Weight curves of children living in the West of Turkey reached those of their European peers, after 11 years of age in boys and after 12 years of age in girls. At all ages, girls and boys in our region had the lowest weight values. Between 7 and 11 years of age, the median height in boys and girls were similar in the West of Turkey and in Europe. At older ages, median height was higher in Turkey. Girls and boys living in Eastern Turkey were the shortest children until 16 years of age; after that age, their height was similar to their peers.

**Conclusions:** Weight may interact with environmental factors, but genetic potential appears to be the most important factor determining height at 17 years of age. Growth patterns of children should be evaluated using specific reference values for specific regions.

## INTRODUCTION

Growth charts are essential tools used in the assessment of health in children (1,2,3). Standards established in children aged 6-18 years, expressed as percentile curves, are presently in use in Turkey (4,5). There are also reports on growth references for Turkish children who are living in other parts of Europe (6,7). In this paper, we present growth charts derived from cross-sectional height and weight data obtained on a sample of 6 917 school children aged 7-17 years living in Eastern Turkey. Growth patterns of children and adolescents from the same geographic background but reared in different environments were compared. We aimed to evaluate the effect of environmental factors on child growth.

## METHODS

The study protocol was approved by the Ethics Committee of Yüzüncü Yıl University in Van, an eastern province in Turkey and the administrative body of the local educational authority. The growth data were obtained from 6 917 school children in Van city within a six-month period. Students and schools were selected by the stratified sampling method according to socio-economic levels in accordance with the criteria advanced by Hollingshead et al (8). Children with known growth disorders or chronic diseases and those who were receiving any kind of medication were excluded from the study. Only children whose height and weight were between the 3rd and 97th percentiles were included in the study sample. A total of 3 874 girls and 3 043 boys were enrolled. Chronological age was calculated as the decimal age by subtracting the birth date from the observation date. Written consent was obtained from the parents prior to the study and all procedures followed were in accordance with those outlined in the Declaration of Helsinki.

**Measurements**

All measurements were performed by pediatricians or by medical students trained for the technique. Weight and height were measured twice and the average was recorded. The measurements were performed with the children dressed in light underclothing and wearing no shoes. Weight in kilograms (kg) was measured to the nearest 0.1 kg with an electronic scale (Tefal sense, France). Height measurements were taken with the child/adolescent in the erect position without shoes with heels, buttocks and the occiput in the Frankfurt plane against a vertical portable scale (Seca GMTH&CO, 22089 Hamburg, Germany). Body mass index (BMI) was calculated in all subjects by dividing weight (kg) by height (m) squared.

**Statistical Analysis**

Construction of percentile curves was performed with the LMS Chart Maker Pro version 2.3 software program (The Institute of Child Health, London) which fits smooth percentile curves to reference data. The LMS method summarizes percentiles at each age based on the power of age-specific Box-Cox power transformations that are used to normalize data. These three quantities depend on age. The final curves of percentiles are produced by three smooth curves representing L (lambda, skewness), M (mu, median) and S (sigma, coefficient of variation). The LMS transformation equation is: X=M (1+ LSz)1/L) L≠0 or X=M exp (Sz) L≠0 where X is the physical measurement and z is the z-score that corresponds to the percentile. The key task of the transformation is to estimate the parameters L, M and S. With estimates of L, M and S, values of X are connected to the values of z through the above equation (9). The percentile is obtained from a normal distribution table, where the z-score corresponds to the percentile of interest. Descriptive statistics for each whole year (e.g. 7.00-7.99 years, etc.) for each sex were calculated by SPSS version 13.0 (Chicago, IL, USA).

The population between ages 7-17 in this region is reported as 128 000 and the universe of the study included 107 000 subjects. The sample size was calculated with 95% confidence interval using the following equation based on the d=0.1 value taking a standard deviation value of 4.4 for a population meeting the inclusion criteria: n=N.Z2.J2/d2 (N-1)+Z2.J2 (10). Sample size was kept at large given the long period of the study.

Descriptive statistics for each age group were studied using SPSS 13.0 (Chicago, IL, USA). Pearson’s correlation coefficient was used to evaluate the linear relationship among weight and height with age for both genders. Student’s t-test was used to determine the differences between countries/regions in terms of weight, height and BMI.

## RESULTS

**Figure 1:**
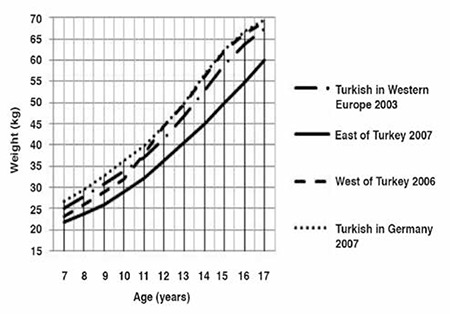
Weight-for-age in Turkish boys born and reared in different geographical regions (median values)

**Figure 2:**
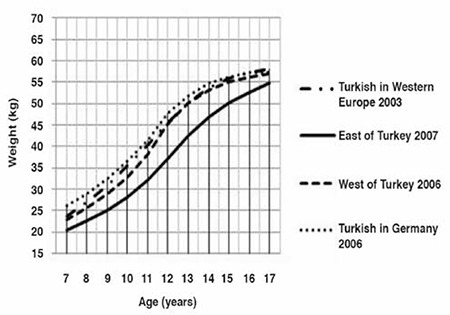
Weight-for-age in Turkish girls born and reared in different geographical regions (median values)

**Figure 3:**
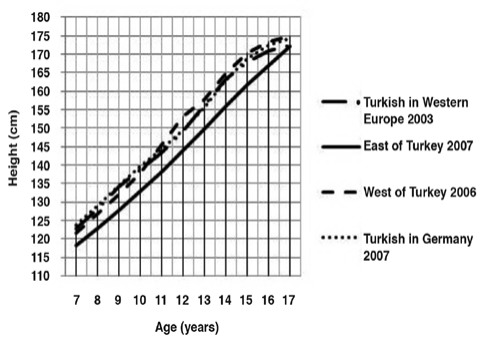
Height-for-age in Turkish boys born and reared in different geographical regions (median values)

**Figure 4:**
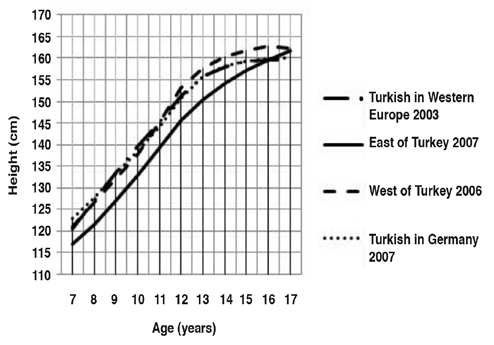
Height-for-age in Turkish girls born and reared in different geographical regions (median values)

**Figure 5:**
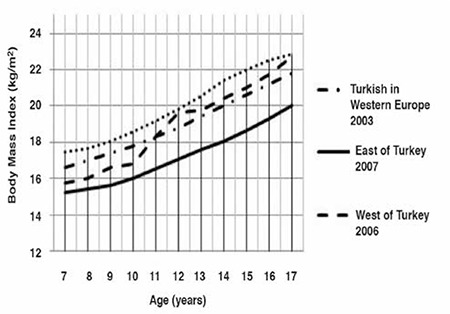
Body mass index-for-age in Turkish boys born and reared in different geographical regions (median values)

**Figure 6:**
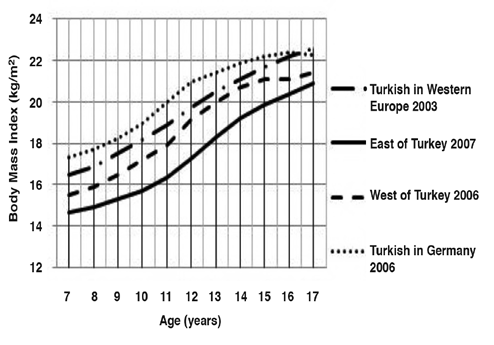
Body mass index-for-age in Turkish girls born and reared in different geographical regions (median values)

The median and standard deviation values for weight, height and BMI by age and gender are presented in Table 1. Comparative data on Turkish children reared and living in Eastern Turkey, in Western Turkey and those born and living in Western Europe and Germany are given in Figures 1 to 6.

**Weight-for-Age**

Between 7 and 11 years of age, the boys living in Turkey (Eastern and Western) were lighter than their European peers (Figure 1). After age 11 years, weight curves of boys living in Western Turkey and of those living in Germany were superimposed and thereafter these two groups continued to be the heaviest of all. The differences between Eastern and Western Turkey increased with age. The boys living in Eastern Turkey were consistently lighter than their peers at all ages. The median weight curve of children born and reared in Western Europe was in the center. The boys in Germany were the heaviest at all ages, with a difference of at least 5 kg.

Girls presented similar patterns to those observed for boys (Figure 2). Between 7 and 15 years of age, the girls living in Germany were the heaviest. Weight curves of girls living in the West of Turkey and those in Western Europe were superimposed at ages 12-15 years. At 15 years of age, all three curves were superimposed on each other. The girls living in Eastern Turkey were the lightest at all ages. The differences in weight for age were found to increase up to 12 years of age, then showed a decrease and were lowest (2.4 kg) at 17 years of age.

**Height-for-Age**

Until 11 years of age, the boys living in the West of Turkey were shorter than their European peers, but they were taller between 11 and 16 years of age (Figure 3). The boys living in the East of Turkey were shorter than their peers at all ages (until 17 years of age). However, the four curves were very close at 17 years of age. The boys living in the West of Turkey were taller than their European peers at 17 years of age (by about 2 cm).

Until 12 years of age, the median height curve of girls living in the West of Turkey was equal to those of their European peers (Figure 4). After that age, these girls were the tallest of all. Height of Turkish girls living in Eastern and Western Europe were very close to one another at all ages. Only at 7 and 11 years of age, the girls in Western Europe were somewhat taller. Until 16 years of age, the girls living in the East of Turkey were shorter than their peers. Again as seen in boys, the four curves were very close to one another at 17 years of age. At the end of 17 years of age, height curves of girls from Eastern and Western Turkey were equal to one another and higher than others.

**BMI-for-Age**

The boys living in Turkey had a lower BMI than those living in Europe, between 7 and 11 years of age (Figure 5). The difference increased with age. The curves of boys living in the West of Turkey and in Western Europe were almost overlapping after 12 years of age, but this value was somewhat higher in Western Turkey. Turkish boys living in Germany had the highest BMI, while those living in the East of Turkey had the lowest, at all ages. The difference increased from 7-to-12 years of age and thereafter became smaller.

The patterns observed for boys were also evident in girls (Figure 6). Curves of girls living in the West of Turkey and Western Europe were overlapping between 12 and 16 years of age, although the curve was somewhat higher in girls in Western Europe. The greatest difference was observed at 17 years of age. The girls in the region had the lowest BMI values and those living in Germany had the highest.

## DISCUSSION

The current paper presents the mean and standard deviation values of height, weight and BMI of Turkish children aged 7-17 years living in the East of Turkey. We compared these data with the data of their peers living in other regions of Turkey and in other countries. The boys and girls living in Eastern Turkey were shorter, lighter and had lower BMI values when compared to their western peers at all ages. The Eastern regions of Turkey are quite different from the Western ones in terms of climate, geographical conditions, socio-economic level, education and nutrition. These factors as well as emotional factors may have an effect on growth in childhood. Our data also shows that environmental factors have an impact on weight and BMI, particularly at ages younger than 17.

The literature on growth references generally has focused on the growth data of children of same geographic background and their comparison with other countries (11,12). There are a number of studies in which growth references for children of different races living in the same community were constructed and compared with each other (6,7). On the other hand, studies on growth patterns of children of similar background but living in different geographical regions or of socio-economic status are scarce (3).

In this article, Turkish children reared in different geographical regions or countries are compared. When we searched the literature, we were not able to find a study as comprehensive as ours.

In childhood and adolescence, standard reference values are used to evaluate growth and nutritional status. Monitoring individual growth is also important for public health. The growth of healthy children of different ethnic backgrounds and living in different countries are usually similar until mid-childhood; therefore, the international reference values are applicable globally for assessing growth and nutritional status in early childhood (13,14). However, it is known that there are marked differences in final heights among countries, even among those that are equally well off and also geographically close to one another (15). It is also known that the anthropometric values used to assess children’s growth are influenced by genetic and environmental factors. In developing countries, growth retardation, infection and malnutrition in children are mostly caused by environmental factors (16,17).

Currently, percentile curves that were updated in 2006 are used for monitoring the growth of Turkish children. Large differences still exist between Eastern and Western Turkey, since the West is more developed with respect to environment, socio-economic level, educational status, life habits and diet. These differences are reflected in the growth status of the children.

The size of the Turkish population living in Europe has increased over the past years as a result of large numbers of families who have emigrated to Europe from every region of Turkey. Today, there are third- or fourth-generation Turkish children being raised in Europe. Turkish children living in Europe and in Turkey have the same geographic background, but their environment, socio-economic level, lifestyle and diet are quite different. Recent publications indicate that Turkish children and adolescents born in Europe show differences in growth from German or Dutch children (6,7).

Children and adolescents of both genders in Eastern Turkey were lighter than their peers else where, at all ages. This finding may be associated with the large family structure and low socio-economic status in Eastern Turkey. Boys and girls in this region were also shorter than their peers, but they caught up with them at 17 years of age in boys and at age 16 in girls. The difference in median height between Eastern and Western Turkey has decreased to 1 cm in girls at 17 years of age. These girls were also taller than their European peers. On the other hand, constitutional short stature and delayed puberty is commonly seen, particularly in the boys living in Eastern Turkey. Thus, if the study could have been extended to cover males up to 19 years, it might have been possible to show that male adolescents in both regions of Turkey are of an equal height.

In conclusion, our study shows that weight and BMI may interact with socio-economic status, dietary habits and maybe medical care, but that these variables do not appear to affect height at 17 years, possibly indicating the role of a genetic potential. However, differences in nourishment and socio-economic level may result in delayed puberty and a late growth spurt. Consequently, the peak growth velocity may shift to more advanced ages. This will lead to different growth patterns in children of a similar geographic background but reared in different environments. Growth patterns of children should be evaluated using specific reference values for specific regions.

**Acknowledgements**

We would like to thank Novo Nordisk Company for supporting this work by providing the measurement devices. We also thank Neyzi O, Bundak R, Fredriks AM and Redlefsen T who let us use their publications for comparing the median values.

## Figures and Tables

**Table 1:**
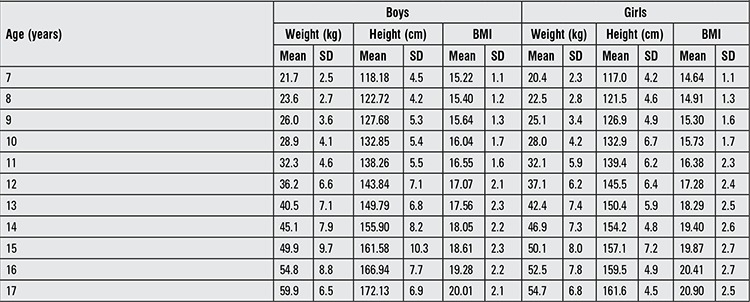
Mean and standard deviation values for height, weight and body mass index (BMI) in children/adolescents included in the study
